# Underground Adaptation to a Hostile Environment: Acute Myeloid Leukemia vs. Natural Killer Cells

**DOI:** 10.3389/fimmu.2016.00094

**Published:** 2016-03-09

**Authors:** Nicolas Dulphy, Anne-Sophie Chrétien, Zena Khaznadar, Cyril Fauriat, Arash Nanbakhsh, Anne Caignard, Salem Chouaib, Daniel Olive, Antoine Toubert

**Affiliations:** ^1^UMRS-1160, Institut National de la Santé et de la Recherche Médicale (INSERM), Paris, France; ^2^U 1160, Université Paris Diderot, Sorbonne Paris Cité, Paris, France; ^3^Laboratoire d’Immunologie et Histocompatibilité, Hôpital Saint-Louis, Assistance Publique-Hôpitaux de Paris (AP-HP), Paris, France; ^4^Centre de Recherche en Cancérologie de Marseille (CRCM), Equipe Immunité et Cancer, INSERM, U1068, Institut Paoli-Calmettes, Aix-Marseille Université, UM 105, CNRS, UMR7258, Marseille, France; ^5^U 753, INSERM, Villejuif, France

**Keywords:** natural killer cells, acute myeloid leukemia, immunoediting, natural killer receptors, immune escape of cancer, aging and cancer

## Abstract

Acute myeloid leukemia (AML) is a heterogeneous group of malignancies which incidence increases with age. The disease affects the differentiation of hematopoietic stem or precursor cells in the bone marrow and can be related to abnormal cytogenetic and/or specific mutational patterns. AML blasts can be sensitive to natural killer (NK) cell antitumor response. However, NK cells are frequently defective in AML patients leading to tumor escape. NK cell defects affect not only the expression of the activating NK receptors, including the natural cytotoxicity receptors, the NK group 2, member D, and the DNAX accessory molecule-1, but also cytotoxicity and IFN-γ release. Such perturbations in NK cell physiology could be related to the adaptation of the AML to the immune pressure and more generally to patient’s clinical features. Various mechanisms are potentially involved in the inhibition of NK-cell functions in AML, including defects in the normal lymphopoiesis, reduced expression of activating receptors through cell-to-cell contacts, and production of immunosuppressive soluble agents by leukemic blasts. Therefore, the continuous cross-talk between AML and NK cells participates to the leukemia immune escape and eventually to patient’s relapse. Methods to restore or stimulate NK cells seem to be attractive strategies to treat patients once the complete remission is achieved. Moreover, our capacity in stimulating the NK cell functions could lead to the development of preemptive strategies to eliminate leukemia-initiating cells before the emergence of the disease in elderly individuals presenting preleukemic mutations in hematopoietic stem cells.

## Introduction

In recent years, the field of cancer immunology has known a growing interest due to development of innovative therapeutic strategies in various malignant pathologies. Since the first hypothesis by P. Ehrlich at the beginning of the twentieth century suggesting that the organism could defend itself against tumor cells (1), through the “Immunosurveillance” theory developed by Burnett (2) and Thomas (3) in the late 1950s, and into the more recent “three Es of the immunoediting” suggested by Schreiber et al. (4); scientists and clinicians learnt that not only cancers were capable of inhibiting the tumor-specific immune response but also host immune cells could potentially be restored or manipulated to eliminate tumors cells. Therefore, therapeutic strategies combining conventional chemotherapy treatments and reinforcement of the self anticancer immunity appear as very promising. Recent successes in the use of immune checkpoint inhibitors to restore the T-cell response against solid tumors are in favor of such approaches (5). Interestingly, the observation that immune cells need external interventions to recover an activity against the autologous tumors demonstrates, as a negative, the adaptation process engaged by tumor cells in order to expand despite the patient’s immune system. Leukemic diseases are particularly suitable to study the dialog with the immune system, as they develop in the same bone marrow (BM) environment as normal hematopoiesis, are well molecularly characterized and also because they invade the organism through the circulation network, so directly in contact with the circulating immune cells.

## Acute Myeloid Leukemia: A Long-Term Malignant Process, Side Effect of Aging

Acute myeloid leukemia (AML) is a heterogeneous group of diseases characterized by the proliferation of a hematopoietic progenitor clone blocked in its differentiation (6, 7). The blockage can concern each maturation step of the myeloid precursors, including granulocytic, monocytic, megakaryocytic, and erythroid precursors. In general, the disease develops in the BM, and the presence of malignant clones inhibits the normal hematopoiesis not only by reducing space available for healthy hematopoietic stem cells (HSCs) but also by direct inhibition (8). This inhibition leads to marrow failure associated with cytopenia. The annual overall incidence of the disease is 3.8 cases per 100,000 adults in western countries, but it increases to 15 cases per 100,000 for elderly over 60 years (9). Many advances have been made in the molecular characterization of the disease and the evaluation of molecular markers in specific cytogenetic AML subsets is now a standard procedure for patient’s diagnosis and risk stratification (9, 10). Moreover, attempts were also developed to categorize AML based on mutation profiling (11–13) or on gene expression profiling, associated or not with recurrent acquired mutations identified in routine diagnosis (14–17). Finally, a new paradigm is taking form in our understanding of the connection between aging and leukemia with the identification of recurrent mutations in genes involved in the epigenetic regulation of the HSCs genome (*DNMT3A*, *TET2*, and *ASXL1*), acquired with age in healthy HSC, and leading to clonal hematopoiesis associated with increases in the risk of hematological cancer, including AML (18–20). The demonstration that healthy HSC could integrate mutations originally identified in AML is coherent with the identification of leukemia stem cell (21), with the potential to initiate a malignant clone at the origin of the disease. Indeed, preleukemic HSCs, defined as a pool of HSC with recurrent *DNMT3A* mutations but without the additional mutations observed in AML blasts, were found in AML patients (22).

Altogether, these observations are in favor of the hypothesis that HSCs accumulate somatic mutations and give rise to AML-initiating cells following a clonal selection process (23) at diagnosis and also after relapse (24). This long duration of the malignant development process, in parallel with patient’s aging, questions the nature of the stimuli leading to this evolution, why particular successive mutations are required to ensure AML survival and proliferation, and how the organism’s environment, including the immune system, can deal with the emerging preleukemic and leukemic cells.

## The Natural Killer Cell: A Major Antitumor Effector Cell

Among the different immune partners, natural killer (NK) cells were defined, at the time of their discovery, as being capable to directly eliminate tumor cells (25–28). NK cells are lymphocytes from the innate immunity, therefore characterized by the absence of rearranged antigen-specific receptors, such as T-cell or B-cell receptors. This population was recently assigned to a newly described family of innate lymphocytes, comprising various innate lymphoid cells (ILCs) (29). Innate lymphocyte populations show some analogies with the subdivision observed for the T-lymphocytes family with the CD8^+^ cytotoxic T-cells, and the Th1, Th2, and Th17 CD4^+^ T-cells. Similarly, conventional NK cells constitute the cytotoxic innate lymphocytes with capacities to eliminate infected or transformed target cells, whereas ILC subsets are capable to support the development of the local immune response through the production of cytokines, such as IFN-γ (ILC1 subset), IL-5 and IL-13 (ILC2 subset), or IL-17 and/or IL-22 (ILC3 subset). NK cells were first categorized as type 1 cells such as Th1 cells because of their capacity to produce IFN-γ, but the expression of perforin and granzymes authorized to distinguish the cytotoxic ILC, i.e., the NK cell subsets, and the helper ILC1 (30). This role sharing could suggest that innate and adaptive lymphocyte populations can interact and support each other to initiate and sustain the immune response (31).

Natural killer cells represent 5–10% of the blood lymphocytes. Two major NK cell subsets are present in blood and secondary lymphoid organs (32). The CD56^dim^CD16^+^ NK cells constitute the vast majority of NK cells in blood (90–95%). They are highly cytotoxic but can also produce significant amounts of cytokines, such as IFN-γ and TNF-α, after stimulation by a sensitive target (33). The expression of the FcγRIII CD16 ensures the capacity for NK cells in mediating the antibody-dependent cellular cytotoxicity (ADCC). By contrast, the CD56^bright^CD16^low/−^ NK cell subpopulation is mainly found in lymph nodes whereas they represent about 10% of blood NK cells (32, 34). The CD56^bright^ NK cells store less intracellular cytolytic vesicles containing perforin and granzymes than their counterpart, but they can secrete large amounts of cytokines in response to an inflammatory environment (32). In addition to the cytokine-mediated triggering, NK cell functions are regulated by a balance between inhibitory and activating signals provided through regulatory receptors on the cell surface (35).

### NK Cell Functions Are Tightly Regulated

Natural killer cells are tightly regulated by numerous receptors that either trigger or inhibit the cell’s functions. To allow the distinction between healthy and abnormal cells (i.e., infected or tumor “stressed” cells) is the ultimate goal of this balance. Indeed, NK cells detect modified target cells that display perturbations in the expression of surface ligands (35).

Through the recognition of some HLA class-I molecules on the target cell, receptors, such as some of the killer immunoglobulin-like receptors (KIRs) or the lectin heterodimer CD94/natural killer group 2, member A (NKG2A), inhibit NK cell functions. Originally described as the “Missing self” theory (36), the physiological function of these receptors is to detect loss or reduction of the class-I antigen-presenting molecules on the surface of tumor cells, a frequent alteration observed in cancer cells (37) and viral infections (38) at the origin of the escape from T-cell-mediated immunity. The absence of HLA class-I molecules on the target cell surface will therefore lead to an absence of inhibition of the NK cell functions. However, chronic exposure of NK cells to HLA class-I loss tumor variants can also lead to NK cell anergy as an escape mechanism. Such anergy can be reversed in presence of IL-12 and IL-18 (39).

Optimal NK cell triggering will also require activation signals provided by activating receptors that detect ligands on the target. A majority of cancers of all cell types express, at variable levels, stress-induced molecules, including the MHC class-I-related chains A and B (MICA/B) and the UL16-binding proteins (ULBPs) (40). These proteins are recognized by the activating lectin-homodimer NK group 2, member D (NKG2D) receptor on NK cells, resulting in the elimination of the tumor (41). Importantly, ataxia telangiectasia, mutated (ATM), and ATM-and Rad3-related (ATR) protein kinases activation as a response to DNA damage can stimulate NKG2D-ligands (NKG2D-L) surface expression (42). Other pathways regulate the expression of certain NKG2D-L [reviewed in Ref. (43)] and participate in leukemic physiology, including alterations in the microRNAs repertoire (44), the heat shock stress pathway (45), which can induce MICA and MICB gene expression, or the activation of the PI3K pathway that is often constitutively activated in leukemia (46) and can stimulate the expression of the mouse NKG2D-L RAE-1. Stress signals associated with DNA damage response, including reactive oxygen species (ROS), can also promote the expression of the poliovirus receptor (PVR or CD155) recognized by the activating receptor DNAX accessory molecule-1 (DNAM-1) (CD226) (47, 48). Interestingly, the DNAM-1 ligands PVR and Nectin-2 were observed on many cancers and DNAM-1 can collaborate with other activating NK cell receptors to mediate killing of tumor cells (49, 50). The natural cytotoxicity receptors (NCRs), such as NKp30, NKp44, and NKp46, were also implicated in the recognition of tumors and notably AML (51, 52) even if ligands are expressed at low levels (53). B7-H6, a ligand for NKp30, and MLL5, from which a short isoform is recognized by NKp44, can be expressed on AML blasts (54, 55). AML cells are also recognized by NKp46 (56), but the ligands involved have not yet been identified. Additional receptors or coreceptors can induce NK cell activation in a cell-to-cell interaction with a target, including the adhesion molecule lymphocyte function-associated antigen-1 (LFA-1), and the signaling lymphocytic activation molecule (SLAM) family receptors, such as 2B4 (CD244), CRACC (CD319), or NTB-A (57). LFA-1 will bind to the intercellular adhesion molecules-1 (ICAM-1 or CD54) expressed on most AML cells (56). The SLAM receptors will be involved in homotypic interactions, except 2B4 (CD244), which will recognize CD48. To date, CD48 was the only SLAM family receptor frequently observed on AML cells (52).

### A Direct Role for NK Cells in the Antitumor Immune Response

The original identification of NK cells as tumor killers has been abundantly confirmed in a wide variety of cancers. The higher susceptibility of murine models lacking NK cells to spontaneous or carcinogen [methylcholanthrene (MCA)]-induced tumors was in favor of a direct role of these lymphocytes in the elimination of malignant cells (4). Numerous *in vitro* and *in vivo* models demonstrated the direct implication of perforin together with IFN-γ produced by cytotoxic cells, including NK cells (58, 59), or of the activating NK receptors (41, 50, 60). In addition, the death receptor pathways involving the Fas-ligand (FasL) receptor and the TNF-related apoptosis-inducing ligand (TRAIL), a member of the TNF family, both induced on NK cells by either IFN-γ or IFN-α/β, were also involved in the antitumor function of NK cells (61, 62). In human, an indirect evidence for a role for NK cells *in vivo* came from the observation of the association between the natural cytotoxicity quantified in blood and the risk of cancer (63). The positive correlation between solid tumor infiltrating NK cells and good prognosis also suggests that NK cells could directly eliminate tumor cells *in vivo* (4). Importantly, in addition to a direct cytotoxicity, NK cells promote the antitumor response through the production of IFN-γ, which is required for the early phase of Th1 priming and polarization in the lymph node (64) and also for the stimulation and polarization of macrophages (65).

## AML Escape from NK Cells: Immediate and Long-Term Processes

**Figure 1:**
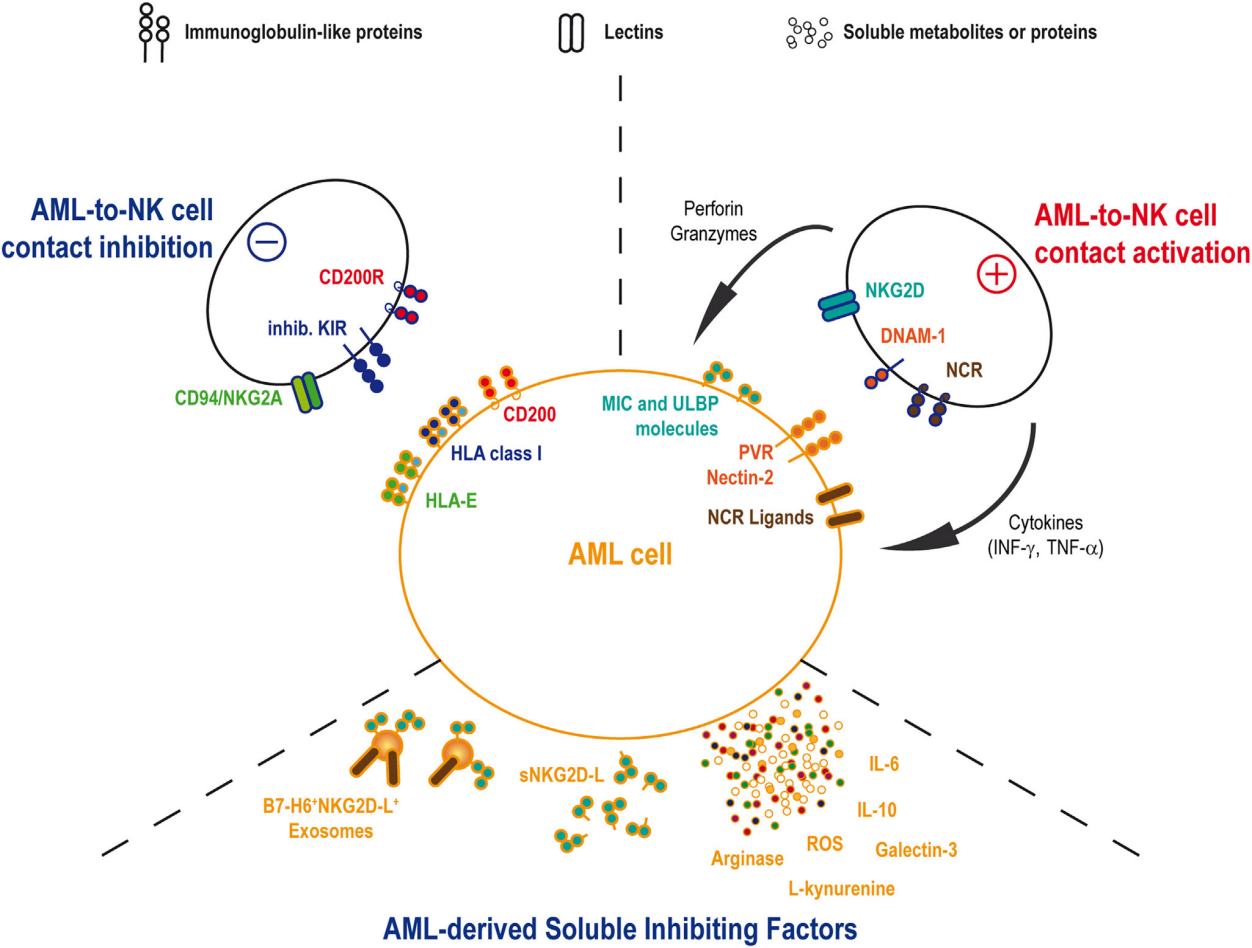
**AML and NK cells interactions**. AML and NK cells will interact by cell-to-cell contact as well as soluble factors. NK cell functions, including cytotoxicity and cytokines release, will be activated through the binding of activating receptors onto the cognate ligands on AML blast. Conversely, AML cell will attempt to inhibit NK cell functions by mobilizing ligands for inhibitory NK receptors. In the same time, AML will secrete some inhibitory soluble agents in order to reduce NK cell efficacy to detect and eliminate leukemia.

*In vivo* AML sensitivity to NK cell-mediated cytotoxicity has been shown by Ruggeri et al. in patients treated by haploidentical HSC transplantation (66). This team reported a lower incidence of relapse in patients transplanted with graft containing alloreactive NK-cell clones against recipient cells. By contrast, the absence of such NK-cell incompatibility was associated with a high relapse rate (66, 67). This observation was based on the existence of KIR/KIR-ligand (i.e., HLA class-I molecules) mismatches in the responsive donor/recipient pairs. The absence of cognate ligands for the inhibitory KIRs allowed NK cell activation by AML cells and elimination of the leukemic target. This illustration of the “missing-self” theory (36) found its counterpart in the observation that the activating KIR2DS1 can also provide a significant reduction of AML relapse in donor/recipient pairs where recipient expresses specific HLA-C ligands (68). According to these results, the selection of the donor may be of importance in order to optimize the graft-vs.-leukemia effect expected from the HSC transplantation. Therefore, haploidentical and umbilical cord blood transplantations would be the most suitable transplantation settings to find KIR/KIR-ligands mismatches. A few studies analyzed the role of KIR/KIR-ligand mismatch in cord blood transplantation setting with contradictory results either demonstrating the advantage of such treatment in myeloid leukemia (69) or, by contrast, showing a higher risk of acute graft-vs.-host disease without curative advantages after reduced intensity conditioning (70). Cytomegalovirus (CMV) infection or reactivation in transplanted patients could explain these discrepancies, as CMV-driven NK cell expansion and maturation could participate in the reduction of the relapse risk (71). Clinical trials using infusions of IL-2-activated haploidentical NK cells in AML patients showed encouraging results with *in vivo* expansions of donor NK cells and complete remissions (CR) in certain patients, suggesting an antitumor NK-cell-mediated immune response (72, 73). However, patient’s autologous NK cells often show defects at diagnosis. Activating receptors, such as DNAM-1, and the NCR, such as NKp30 and NKp46, present reduced expression levels on NK cell surfaces in a large proportion of patients (74–76). The inhibitory receptor CD94/NKG2A can also be upregulated on patient NK cells (77). In parallel to the phenotypic perturbations, cytotoxic activity and IFN-γ release are also decreased (76–78). These defects were associated with pejorative outcomes, including increased relapse risk and/or reduced overall survival. Interestingly, NK cell phenotype and function are normalized after chemotherapy treatment, underlying the role for AML blasts in decreasing NK cell’s abilities (75, 76). Altogether, these observations imply that AML inhibits the autologous NK cell response through several mechanisms (Figure 1).

### Leukemogenesis and Immune Escape

Tumor microenvironment plays a critical role in the inhibition of the antitumor immune response (79). Leukemogenesis occurs mainly in the BM, the primary site for the healthy hematopoiesis. This suggests that leukemia BM environment could modify the immune cell differentiation process and also that healthy immune populations may influence leukemogenesis. Such interference was demonstrated in myelodysplastic syndromes (MDS), a heterogeneous group of myeloid disorders displaying low to high risk to give rise to secondary AML (80). As observed in AML patients, blood NK cells from MDS patients show severe defects with downregulation of activating receptors, including NKG2D and DNAM-1 (81), reduced cytotoxic activity (81, 82), and increased apoptosis rate in response to IL-2 stimulation associated with reduced proliferation *in vitro* (82). Importantly, increased NK cell defects were also associated with high-risk MDS, characterized by higher International Prognostic Scoring System (IPSS) Score, presence of excess blasts, abnormal karyotype, and hypercellularity (83). BM NK cells in MDS patients are also deeply affected by the disease, suggesting that MDS BM environment could play a role in those defects (81–83). In AML, BM environment influences healthy hematopoiesis by affecting BM cell populations. Notably, BM stromal cells show low proliferative rate as well as genetic and epigenetic alterations (84). Similar numbers of healthy CD34^+^CD38^−^ HSC were found in the BM of AML patients and healthy individuals. However, normal CD34^+^CD38^+^ progenitors were found reduced in BM of AML patients likely resulting from a differentiation block at the HSC-progenitor progression (8). Consequently, similar to MDS, NK cell differentiation in the BM seems to be affected by AML. A recent work by Vasold et al. suggested a role for mesenchymal stromal cells and hypoxia in the reduction of NK cell cytotoxic activity against autologous AML blasts highlighting the importance of the BM stroma in the emergence of abnormal mature immune cells in the peripheral blood (85). Connected to this observation, we recently described that AML cell transcriptional program is intimately associated with the deepness of the NK cell defects (76). NK cell deficiencies were associated with an increased risk of relapse. AML blast transcripts coding for proteins involved in cytokine/cytokine receptor and chemokine pathways were found severely diminished in patients with defective NK cells. In return, IFN-γ production by CD56^bright^ NK cells is almost abrogated at diagnosis in those patients (76). Altogether, these observations would indicate that AML blasts can modify BM environment, including stromal, precursor, and mature immune cell populations. In addition, the immune pressure, notably provided by NK cells, could influence the AML transcription program leading to AML escape and a more pejorative outcome.

### Cell-to-Cell Contact and Defective Immunological Synapse

Interactions between immune cells and targets constitute a multistep process where first immune cells build conjugates with the target partner and then initiate a reorganization of proteins localized at the membrane at the cell-to-cell contact point [reviewed in Ref. (86)]. This includes actin mesh polymerization and centrosomes polarization (87). The supramolecular activation cluster (SMAC) recruits regulating NK receptors, in parallel with ligands on the target cell, together with costimulatory and adhesion molecules in order to integrate and amplify intracellular signaling. NK cells will therefore polarize cytolytic vesicles toward the immunological synapse (IS) (i.e., the target cell) and secrete perforin and granzymes within the intercellular space (86). At the resolution of the synapse, NK cells express low levels of activating receptors and need a period of time to regain their full function (88).

Even if leukemic cells are sensitive to allogeneic NK-cell-mediated lysis, alterations in the expression of some activating receptors, including NCR and DNAM-1, on the autologous NK cells suggest that NK-to-AML interactions can be defective in patients. Such observation has been made for T cells with a reduction in actin polarization and phosphotyrosine signaling in T-cell/AML blast conjugates (89). Reduction of NKR expression by autologous NK cells was partially associated with the presence of cognate ligands on AML cells as *in vitro* coculture of NK cells with AML cell lines or primary blasts decreases the NKR levels (56, 90). However, this phenomenon is not proportional to the ligand expression levels on the target, suggesting additional mechanisms controlling the profound NKR downregulation observed *in vivo*. When studying the IS between NK and AML cells, NK cells showed defects in the polarization of their cytolytic granules toward the IS against AML blasts (56). Consequently, defects in NKR expression may also be the result of a continuous exposure to ligands in incomplete cytotoxic synapses against AML blasts, leading to an exhaustion of the NK-cell cytotoxic activity. Therefore, NK cell cytotoxic activity is progressively switched off, whereas AML cells survive and proliferate. Whether the decrease in IFN-γ production observed in patients is also the result of perturbations in the IS or in downstream NKR signaling pathways requires further studies. The molecular mechanisms responsible of this cytotoxic defect need also to be identified. Inhibitory KIRs can reduce the autologous anti-AML response as demonstrated in autologous HSC transplantations (91). Nevertheless, inhibitory KIRs likely play a very early role in the building of the IS during polymerization of the actin network and would be only marginally implicated at later stages to inhibit the lytic granules’ polarization (86). Other receptors could be good candidates in the negative regulation of NK functions, such as CD96, as demonstrated by the higher resistance of *Cd96*^−/−^ mice to solid tumors (92). On the other side, CD200 (also named OX2) expressed at high levels on some AML has been recently identified as a suppressor of patient’s NK cell cytotoxic and cytokine secretion functions in the antitumor response (93).

### Soluble Molecules

In addition to cell-to-cell contact-based inhibition, NK cells’ functions are inhibited by various soluble molecules, including soluble ligands of regulatory NK receptors, cytokines, such as TGF-β or IL-10, metabolic compounds, such as ROS, and tryptophan or arginine catabolites.

Some AML clones have adopted strategies to inhibit NK cells with specific soluble compounds. To date, NKG2D and NKp30 were the main targets described for such inhibition. Soluble ligands can be released by tumor cells into the extracellular environment. NKG2D-ligands (NKG2D-L) and the NKp30 ligands B7-H6 are cleaved by metalloproteinases, either matrix metalloproteinases (MMP) or A disintegrin and metalloproteinases (ADAMs) (94–99). Soluble NKG2D-L was found in the serum of patients with solid tumors or hematological cancers. Moreover, soluble MICA release is regulated by chaperones, such as ERp5, modulating MICA structure with the support of the heat shock protein GRP78 to induce conformational changes allowing its cleavage (100, 101). Despite a high heterogeneity in the surface expression of NKG2D-L by AML, the majority of patients shows soluble forms of MICA, MICB, or ULBP2, alone or more often in combination with up to five soluble NKG2D-L (102). As observed in solid tumors (103), the presence of soluble NKG2D-L in AML patients’ serum is associated with a reduction of the surface NKG2D expression leading to a decrease in NKG2D-mediated NK-cell’s activity (102). Importantly, a recent work by Deng et al. showed that the soluble form of MULT-1, a mouse high-affinity NKG2D-L, released by tumors, can stimulate NK-cell cytolytic function, and induce tumor rejection in mice (104). Activating soluble NKG2D-L was not identified in human yet, but such molecule, if it exists, could be of interest for inducing antitumor NK-cell function.

B7-H6 shedding by solid tumors seems to induce a reduction of the NKp30-mediated tumor cell recognition by NK-cells (96). In contrast to soluble NKG2D-L, this process would be due to a reduction of the B7-H6 expression on the tumor cell surface rather than to a direct inhibition of the NKp30 expression on the NK cell. No observation of B7-H6 shedding by AML has been made yet but the expression of B7-H6 on AML cells together with the reduction of expression of NKp30 on patient’s NK cells would justify studying this pathway in AML patients.

Soluble NKG2D and NKp30 ligands can be also released in the serum bound to tumor-derived exosomes (TEX) (105, 106). Exosomes from solid tumors or leukemia/lymphoma cells can present MICA, MICB, and ULBP molecules leading to an inhibition of the NKG2D-mediated NK cell activation (105, 107). In the same way, a reduction of NKp30 can be observed when NK-cells are incubated with B7-H6 positive exosomes produced by myeloid subsets in inflammatory conditions (108). By contrast, exosomes carrying BAG6, another NKp30 ligand, are necessary to activate NK cells in order to eliminate chronic lymphocytic leukemia (CLL) cells, whereas soluble BAG6 lead to tumor evasion (106). In addition, soluble galectin-3 produced by solid tumor cells works as an inhibitory ligand of NKp30 (109). In line with this observation, higher levels of galectin-3 gene expression in BM are an independent unfavorable prognostic factor for overall survival in patients with AML (110). Altogether, these observations would suggest that soluble or exosomes bound NKp30 ligands could also interfere, in parallel with other soluble ligands, with NK cell functions in AML patients.

Imbalance in serum cytokines can be responsible for perturbations in the regulation of the antitumor response. In contrast to reduced TGF-β levels in plasma of AML patients as compared to healthy individuals (75, 111), IL-10 was found significantly higher together with the proinflammatory cytokines IL-6 and TNF-α. Curiously, high levels of IL-6 and low levels of IL-10 are associated with poor outcome (111). Even if such cytokine environment is probably more related to the AML physiology, it still can influence immune cell properties. Indeed, high levels of IL-6 were shown to impair perforin and granzyme B expression and reduce NK cell cytotoxic activity in individuals with autoimmune diseases (112), with heart failure (113), and cancer patients treated with recombinant IL-6 (114).

In parallel, high levels of small immunosuppressive molecules, side products of the leukemic cell metabolism, can be released by AML blasts. ROS participate to NK-cell defects in the expression of activating receptors, such as NKp46 and NKG2D (115). Interestingly, they are highly produced by AML with specific mutation patterns, such as activating mutations in RAS family members or FLT3/ITD mutations (116, 117). Arginine metabolism is also enhanced in AML blasts leading to an immunosuppressive environment. High levels of production of active arginase II by AML blasts can induce an accumulation of this enzyme in the plasma of patients, resulting in significant inhibition of lymphocyte proliferation (118). In addition, the immunoregulatory enzyme indoleamine 2,3-dioxygenase (IDO) is also expressed by leukemic blasts, whereas it is absent from normal hematopoietic CD34^+^ stem cells (119). IDO catalyzes tryptophan degradation by producing l-kynurenine, which can directly affect NK cell phenotype and cytolytic function through the inhibition of the cytokine-induced upregulation of NKp46 and NKG2D (120). In addition, IDO activity can stimulate the emergence of CD4^+^CD25^high^ regulatory T-cells (Tregs) (121) capable of inhibiting NK cell functions by TGF-β release (122) or IL-2 starvation (123).

## AML and Conventional Treatments: Resistance to Chemotherapies

Acute myeloid leukemia treatment by conventional chemotherapy eliminates tumor blasts and leads to the achievement of CR in 70–80% of patients younger than 65 years (6). Elimination of circulating AML blasts allows the recovery of NK cell phenotype and functions (75, 76), and a sustained autologous NK cell activity can support a continued CR (124). However, at least half of patients will eventually relapse (6). A major limitation for success in chemotherapy of AML is dominance of drug-resistant subpopulations of cells. AML cells also can achieve the resistance phenotype through modification of multiple and diverse pathways, such as inactivation of the mitochondrial apoptotic machinery, decreased expression of proapoptotic agents, upregulation of antiapoptotic molecules, and promotion of drug efflux. Although daunorubicin (DNR) is the most efficient and widely used anthracycline to treat AML, resistance to this drug remains a critical problem (125–127). In this regard, the intrinsic and acquired resistance of AML to drug treatment remains a fundamental challenge for improving patient outcome. One of the consequences of acquisition of drug resistance by leukemic cells is the appearance of cross-resistance against immune effector cells. We have recently demonstrated that the acquisition of resistance to DNR resulted in the acquisition of cross-resistance to NK cell-mediated cytotoxicity. MiR microarray analysis revealed that this cross-resistance was associated with miR-181a downregulation and the subsequent upregulation of MAP3K10 and MAP2K1 tyrosine kinases and the BCL-2 (BCL-2 and MCL-1) family. These studies point to a determinant role of miR-181a in the sensitization of leukemic resistant cells to DNR and NK cells and suggest that miR-181a may provide a promising option for the treatment of immuno- and chemoresistant blasts (128).

In contrast, previous studies showed that NKG2D-L can be upregulated on the AML cell surface after treatment with various molecules, including the histone deacetylase inhibitor valproic acid (VPA) (129, 130). In the same way, the administration of all-*trans*-retinoic acid (ATRA) can also increase the NKG2D-L expression levels on acute promyelocytic leukemia, a particular subtype of AML with a *PML-RARA* gene fusion (130). Importantly, such increased expression of NKG2D-L cannot be observed in patients treated with chemotherapy in absence of ATRA or VPA (130). These observations suggest that chemotherapy can stimulate an anti-AML NK-cell–mediated response. Recently, we showed that cytarabine-resistant cells become more susceptible to NK-mediated cell lysis as compared to parental cytarabine-sensitive cells. The increased susceptibility correlates with the induction of ULBP 1/2/3 and NKG2D-ligands on target cells by a mechanism involving c-Myc induction (131). These studies could help to improve the efficacy of NK-cell-based therapy that allows for better designing of NK-based immunotherapy.

## Immunotherapeutic Strategies

Given the sensitivity of AML to NK-cell-mediated lysis, strategies to enhance or restore NK cell functions in patients could be of interest besides conventional chemotherapy. Numerous methods have been developed during the last few years in order to either modulate immunity against tumors using immunomodulatory drugs (IMiDs) or cytokines or to specifically target or activate NK cells against leukemia cells. Such treatments, used alone or in combination with chemotherapy, aim to eliminate chemoresistant tumor cells.

### Immunomodulatory Drugs

Immunomodulatory drugs are structural and functional analogs of thalidomide (132). To date, two molecules have been approved in MDS, multiple myeloma (MM), or mantle cell lymphoma (MCL): lenalidomide and pomalidomide. Alone (for MDS or MCL) or in combination with dexamethasone (MM), as a second or third line of treatment, IMiDs improve both time-to-progression and overall survival of patients. Several studies have also explored the synergistic effect of IMiDs with rituximab for the treatment of CLL (133) or MCL (134). Beyond their direct effect on cancer cell proliferation and angiogenesis, these molecules stimulate antitumor effectors, including B, T, and NK cells (135). Hence, by their broad range of effects, IMiDs represent a novel strategy for immunotherapy as evidenced by numerous ongoing clinical trials, in many cancer settings, including AML (136).

In the case of NK cells, IMiDs increase the expression of activating receptors, notably NCR (137, 138). These molecules induce expansion of NK cells as confirmed by immunomonitoring studies in several clinical trials (137, 139, 140). Enhanced NK cell ADCC or natural cytotoxicity is largely mediated *via* IL-2 produced by T cells (141). In addition, we have recently shown that lenalidomide enhances tumor cell recognition by NK cells by improving the stability of the immune synapse (56). Finally, IMiDs increase tumor infiltration by NK cells in murine models (142).

### Cytokines

Several cytokines of the IL-2 family are essential for NK cell survival, expansion, and activation, but so far, only IL-2 has an approval for clinical use. In the family of IL-2, IL-15 and IL-21 share some characteristics such as activation and proliferation of NK cells, and the common γ-chain for their receptor (143). IL-2 is able to induce expression of NKG2D, NKp44, and NKp46 on NK cells (49, 82, 144). In MDS, however, following *in vitro* IL-2 stimulation, NK cells do not recover a normal cytolytic activity when compared to healthy volunteers (82). Moreover, IL-2 fails to induce NK cell proliferation compared to healthy volunteers, but rather increases the rate of apoptotic NK cells (82). So far, the therapeutic use of IL-2 for the treatment of hematological malignancies has been hampered by a peripheral toxicity (145) and an unwanted expansion of Tregs (146). Conclusions of clinical trials report modest antitumor activity when used as a monotherapy. Therefore, the use of IL-2, especially at high doses, might be restricted to *ex vivo* expansion of NK cells given problems of *in vivo* toxicity (145).

In contrast to IL-2, IL-15 and IL-21 may represent a better alternative because these cytokines do not expand Tregs. Accordingly, many clinical trials currently aim to demonstrate an efficient NK cell-mediated antitumor response with *in vivo* or *ex vivo* IL-15-expanded NK cells in AML. Hence, in the absence of clinical approval for IL-15, several groups are testing the possibility to expand NK cells *in vitro* before reinfusion into patients.

IL-15 plays a major role in the proliferation, differentiation, survival, and functions of T and NK cells (147, 148). *Ex vivo* exposure of NK cells from AML patients to IL-15 enhance NKp30, NKp46, NKG2D, and NKG2C surface expression. Accordingly, this increase of receptor expression correlated with an enhanced natural cytotoxicity against autologous AML cells (147, 149). In addition, in hematological malignancies, low levels of circulating IL-15 after BM transplantation are predictive of risk of relapse (150). In line, NK cell recovery in stem cell transplantation is strongly correlated with plasmatic concentrations of IL-15 (149).

The serum concentration of IL-15 increases dramatically following administration of cytotoxic agents (147, 150). For some authors, this elevation of serum IL-15 could be related to the depletion of lymphoid populations that normally consume circulating IL-15 or to inflammation induced by chemotherapy (149). *In vivo* injections of the IL-15/IL-15Rα heterodimer result in significant expansion of γδ, CD8^+^ T, and NK cells (148). Recently, this cytokine has become available for use in early phase clinical trials as an alternative to IL-2 (147, 148). IL-15 is currently assessed as a therapy for various solid tumors, including refractory metastatic melanoma, metastatic renal cell cancer.

IL-21 has been proven safe in phase I clinical trials with signs of clinical activity (151). IL-21 stimulation of NK cells mainly results in enhanced NK cell functions. *Ex vivo*, IL-21 is capable of inducing NK cell maturation and stimulates the production of IFN-γ and cytotoxic properties of NK cells (152–154). Several clinical trials have reported the effect of IL-21 therapy on immune system after administration in patients with metastatic melanoma and renal cell carcinoma (151). Although NK and T cell numbers were temporarily decreased during administration of IL-21, the cells had higher expression of CXCR3, hyaluronan-mediated motility receptor (HMMR), IFN-γ, perforin, and granzyme. In addition, NK cells from patients displayed an enhanced cytotoxicity capacity (151). These results were confirmed in a phase II trial for metastatic melanoma (155). In the absence of clinical approval, IL-15 and IL-21 are also used *ex vivo* to expand and activate NK cells for further infusion in patients. NK cells stimulated *ex vivo* by the leukemic cell line K562 expressing membrane-bound IL-15 or IL-21 showed a strong proliferation and cytolytic activity with a higher proliferation rate and an increase in telomere length for IL-21-activated NK cells (156). NK cells expanded *ex vivo* by membrane-bound IL-15 are currently infused into MDS or AML patients (phase I clinical trial #NCT02123836).

### Bi- and Trispecific Killer Cell Engager

Several monoclonal antibodies (mAbs) directed against tumor antigens have been generated and are currently used in the clinics. The most famous therapeutic mAbs remains the anti-CD20 rituximab, which is widely used to B-cell-related diseases and cancers. Several mechanisms of action have been identified and one of these is the recognition of the Fc part of the human or humanized IgG1 or IgG3 isotypes by CD16 expressed by NK cells and myeloid cells. Upon engagement of CD16, the cells are activated and kill the targeted cells. Unfortunately, several studies have shown that the polymorphism of CD16 and the engineering process may alter ADCC efficacy. Bispecific killer cell engagers (BiKEs) are engineered antibodies with dual specificity, for a tumor antigen like CD19 or CD20 for B-cell-related diseases and CD16 targeting NK cells. The anti-CD16 part of the BiKEs bypasses the disadvantages of classical mAbs (157). For instance, AFM13, a BiKE targeting CD19 and CD16, has been recently tested in phase I and II trials (#NCT01221571 and #NCT02321592, respectively) in non-Hodgkin lymphoma.

*In vitro* studies demonstrated that CD33 × CD16 BiKEs trigger NK cell activation against AML cell lines and primary targets through CD16 signaling, leading to cytokine and chemokine production (158). As a consequence, significant increases in NK cell cytolytic activity led to induction of target cell apoptosis at high and low target to effector ratios. In a study based on NK cell isolation from patients with MDS, authors showed that CD33 × CD16 BiKE potently activates blood and marrow MDS–NK cells at all disease stages to lyse CD33^+^ MDS and CD33^+^ myeloid-derived suppressor cells (MDSCs) targets (159). Noteworthy, MGD006, a CD123 × CD3 BiKE is tested in a phase I trial (#NCT02152956), confirming the current explosive attention to BiKEs as potent therapeutic tools for AML and other cancers. In the same way, a CD30 × CD16 bispecific tetravalent chimeric antibody (TandAb) was used in a phase I clinical trial in patients with relapsed or refractory Hodgkin lymphoma showing good tolerance and tumor targeting (160). However, with respect to AML treatment, the use of BiKEs remains limited due to the heterogeneity of tumor antigen in this disease.

Recently, several new reagents were developed in attempt to enhance the targeting of malignant cells. Gleason et al. have generated a trispecific mAb (TriKE) directed against CD19, CD20, and CD16 (159). This TriKE, efficiently engaged NK cells against CD19^+^CD20^+^ leukemic targets, as proven by a strong cytotoxicity and IFN-γ production. To increase NK-cell activating properties, Miller et al. have developed a TriKE targeting CD33 and CD16 that contains IL-15 (161). This reagent not only mediates CD16 directed cytotoxicity against CD33^+^ leukemic cells but also sustains NK-cell activation and persistence by the IL-15 linker.

In conclusion, targeted cellular immunotherapy with BiKEs and TriKEs are promising approaches in terms of effector cell retargeting and induction of efficient antitumor response and are currently being developed and evaluated for targeting of various malignancies (162, 163).

### Antibodies Directed against NK Cell Inhibitory Receptors

Among strategies to improve the recognition of tumor cells by NK cells, blocking the inhibitory interactions is appealing. Inhibitory molecules, such as KIR and NKG2A, are expressed at the surface of NK cells and inhibit NK cell activation *via* their ligands (HLA-C and HLA-E, respectively). In the case of cancer patients, expression of KIR and NKG2A, as well as expression of their ligands at the surface of tumor cells, has been described in several solid cancers and leukemias (164–166). Subsequently, activation of NK cells is likely prevented and leads to NK-mediated immune evasion. Inhibition of these mechanisms by blocking antibodies is currently being assessed.

IPH2101 is a fully human IgG4 that blocks the interaction between the major subset of KIR (KIR2DL1, KIR2DL2, and KIR2DL3) and their cognate ligands (167, 168). A second generation of anti-KIR mAb, lirilumab (IPH2102/BMS-986015) with a stabilized hinge was generated (167, 168).

*In vitro* studies showed that IPH2101 augments NK cell-mediated lysis of KIR-ligand matched tumor cells and enhances NK cell-mediated ADCC against antibody-bound tumors (168–170). The therapeutic potential of IPH2101 has also been demonstrated in preclinical mouse models (171, 172), which have formed the basis for clinical trials evaluating IPH2101 in patients with cancer (173). Blocking NK inhibition with the anti-KIR IPH2101 antibody has been proven to be safe in early phase clinical trials in patients with AML and MM (174, 175) and enhances *ex vivo* NK cell cytotoxicity against MM cells (175).

However, a phase II study in MM patients did not reveal lasting objective responses (173). IPH2101 has also been assessed *in vitro* in combination with lenalidomide and potentiates NK-cell cytotoxicity toward autologous myeloma cells. This combination is currently being tested in a phase I clinical trial in MM patients (#NCT01217203) (176). The second generation anti-KIR lirilumab was also shown to synergize with Lenalidomide to increase NK cytotoxicity of myeloma patients treated with Daratumumab (anti-CD38) (177). *In vitro* and *in vivo* lirilumab enhances NK activity against CD20^+^ lymphoma cells (167). With respect to AML, lirilumab is currently tested in patients in CR for long-term maintenance (#NCT01687387), and for the treatment of patients with refractory/relapsed AML (#NCT02399917). Although safe, this therapeutic mAb did not induce impressive clinical improvement so far. First of all, cytotoxic effectors expressing KIRs (NK cells, αβ CD8^+^, and γδ T cells) use other inhibitory KIRs that are not targeted by lirilumab (NKG2A, KIR3DL, and CD85j/ILT2). In an autologous setting, it is likely that these cells may still remain tolerant to leukemic cells. In addition, lirilumab also recognizes KIR2DS1 and KIR2DS2; blocking these receptors may, in contrast, unfavor tumor cell clearance (168).

Noteworthy, a newly engineered mAb directed against NKG2A has been generated by Innate Pharma (IPH2201) and is currently tested in clinical trials (phase I/II) in ovarian cancer, squamous cell carcinoma, and refractory CLL (#NCT02459301, #NCT02331875, and #NCT02557516, respectively). It is tempting to speculate on the efficacy of this new reagent in AML treatment, as these cells are expected to express HLA-E.

Other inhibitory receptors, including PD-1, LAG-3, or TIM-3, usually classified as “inhibitory checkpoint receptors” may influence NK cell activity. The increasing interest for the PD-1/PD-1-ligands axis on T-cells in cancer therapy legitimated the analysis of PD-1 expression on NK cells in various pathological situations. In a mouse model of glioblastoma, NK cell functions against mouse glioma stem cells and the survival of the mice can be ameliorated by blocking either PD-1 or the PD-1 ligand B7-H1 (also named PD-L1) (178). In human, data describing a role for PD-1 in the regulation of NK cells are relatively scarce. Wiesmayr et al. observed the expression of PD-1 on NK cells in pediatric patients with post-transplantation lymphoproliferative disorders caused by EBV infection. The presence of PD-1 was associated with a reduced expression of NKp46 and NKG2D and NK cell function impairment, and blocking the PD-1 inhibitory pathway could restore IFN-γ secretion (179). The PD-1/PD-L1 axis was also involved in the modulation of NK-cell functions against MM (180). Interestingly, IFN-γ can induce the expression of PD-L1 on AML cells leading to the inhibition of the antileukemic response mediated by T-lymphocytes (181) and NK cells (182). Therefore, the anti-PD-1 mAb Nivolumab is tested in a phase II clinical trial in AML patients in remission with high risk of relapse (#NCT02532231).

### Chimeric Antigen Receptor–NK Cells

Another strategy to improve antitumor immunity has arisen from recent advances in cell genetic modification that have allowed the specific targeting of tumor cells by cytotoxic effectors. Most of the tools generated are chimeric antigen receptor (CAR)-T cells, i.e., T cells engineered to express a receptor for tumor antigen (for instance, CD19 in the case of B-cell leukemia) coupled to activate signaling adaptors. The few clinical trials with CAR-T cells have obtained somewhat promising results that should be strengthened by other studies. Genetic modification of NK cells has been more recently performed, but not yet with myeloid tumor specificity. For instance, Töpfer et al. generated NK cell lines or primary NK cells targeting PSCA, a prostate cancer antigen (183). As expected, these cells react against PSCA positive tumor cell lines by secreting IFN-γ and killing these target cells. In line with this observation, several others have been designed, based on NK-92 or other NK cell lines (184–187). This new strategy is promising although the costs may remain a serious limitation.

## Conclusion

Acute myeloid leukemia is the most common myeloid leukemia, usually treated with a combination of anthracyclines and cytarabine in a first attempt to achieve CR. The consolidation phase of the treatment aims to prolong CR and eventually to cure the disease. However, disease heterogeneity (cytogenetic and mutation profile, deepness of BM failure, resistance to treatment) and patient’s general condition (age, secondary AML) led to an unfavorable prognosis for many patients. Over time, AML develops various mechanisms to protect itself from the patient’s immune system and more precisely from NK cells. The long-term coexistence of leukemia-initiating cells, and then tumor blasts, with NK cells, first in the BM and later in the periphery, can explain the emergence of NK cell defects together with immunoresistant AML cells. The antitumor function of NK cells, demonstrated after allogeneic HSC transplantation, justifies developing methods in order to restore, stimulate, or induce NK cell activity in AML patients. Treatments combining the elimination of the peripheral leukemic blasts using conventional chemotherapy, together with the chemoresistant leukemic-initiating cells, targeted by immune mediators, including NK cells, appear very attractive. However, we could consider the opportunity to stimulate NK cell antileukemic functions before the emergence of the disease. Indeed, the recent observation of preleukemic mutations in healthy elderly individuals’ HSC questions the capacity of the immune system to eliminate or tolerate the presence of leukemia-initiating cells in the BM at advanced age. In that context, stimulating the immunosurveillance mediated by NK cells could be a promising preemptive strategy against AML.

## Author Contributions

All the authors substantially contributed to the design of this review. All the authors participated in the writing and the critical review of the draft. All the authors approved the final version of the manuscript and agreed to be accountable for all aspects of the work.

## Conflict of Interest Statement

DO is a founder and shareholder of Imcheck Therapeutics (Marseille, France). The other authors declare no competing financial interests.

## References

[1] Blair GE Cook GP Cancer and the immune system: an overview. Oncogene (2008) 27(45):5868 10.1038/onc.2008.277 18836467

[2] Burnett FM The concept of immunological surveillance. Prog Exp Tumor Res (1970) 13:1–27. 10.1159/000386035 4921480

[3] Thomas L. On immunosurveillance in human cancer. Yale J Biol Med (1982) 55(3–4):329–33. 6758376 PMC2596448

[4] Dunn GP Old LJ Schreiber RD. The three Es of cancer immunoediting. Annu Rev Immunol (2004) 22:329–60. 10.1146/annurev.immunol.22.012703.104803 15032581

[5] Pico de Coana Y Choudhury A Kiessling R. Checkpoint blockade for cancer therapy: revitalizing a suppressed immune system. Trends Mol Med (2015) 21(8):482–91. 10.1016/j.molmed.2015.05.005 26091825

[6] Dohner H Estey EH Amadori S Appelbaum FR Buchner T Burnett AK Diagnosis and management of acute myeloid leukemia in adults: recommendations from an international expert panel, on behalf of the European LeukemiaNet. Blood (2010) 115(3):453–74. 10.1182/blood-2009-07-235358 19880497

[7] Estey E Dohner H. Acute myeloid leukaemia. Lancet (2006) 368(9550):1894–907. 10.1016/S0140-6736(06)69780-8 17126723

[8] Miraki-Moud F Anjos-Afonso F Hodby KA Griessinger E Rosignoli G Lillington D Acute myeloid leukemia does not deplete normal hematopoietic stem cells but induces cytopenias by impeding their differentiation. Proc Natl Acad Sci U S A (2013) 110(33):13576–81. 10.1073/pnas.1301891110 23901108 PMC3746910

[9] Showel MM Levis M. Advances in treating acute myeloid leukemia. F1000Prime Rep (2014) 6:96. 10.12703/P6-96 25374674 PMC4191225

[10] Estey EH. Acute myeloid leukemia: 2014 update on risk-stratification and management. Am J Hematol (2014) 89(11):1063–81. 10.1002/ajh.23834 25318680

[11] Patel JP Gonen M Figueroa ME Fernandez H Sun Z Racevskis J Prognostic relevance of integrated genetic profiling in acute myeloid leukemia. N Engl J Med (2012) 366(12):1079–89. 10.1056/NEJMoa1112304 22417203 PMC3545649

[12] Grossmann V Tiacci E Holmes AB Kohlmann A Martelli MP Kern W Whole-exome sequencing identifies somatic mutations of BCOR in acute myeloid leukemia with normal karyotype. Blood (2011) 118(23):6153–63. 10.1182/blood-2011-07-365320 22012066

[13] Cancer Genome Atlas Research Network. Genomic and epigenomic landscapes of adult de novo acute myeloid leukemia. N Engl J Med (2013) 368(22):2059–74. 10.1056/NEJMoa1301689 23634996 PMC3767041

[14] Mullighan CG Kennedy A Zhou X Radtke I Phillips LA Shurtleff SA Pediatric acute myeloid leukemia with NPM1 mutations is characterized by a gene expression profile with dysregulated HOX gene expression distinct from MLL-rearranged leukemias. Leukemia (2007) 21(9):2000–9. 10.1038/sj.leu.2404808 17597811

[15] Gal H Amariglio N Trakhtenbrot L Jacob-Hirsh J Margalit O Avigdor A Gene expression profiles of AML derived stem cells; similarity to hematopoietic stem cells. Leukemia (2006) 20(12):2147–54. 10.1038/sj.leu.2404401 17039238

[16] Valk PJ Verhaak RG Beijen MA Erpelinck CA Barjesteh van Waalwijk van Doorn-Khosrovani S Boer JM Prognostically useful gene-expression profiles in acute myeloid leukemia. N Engl J Med (2004) 350(16):1617–28. 10.1056/NEJMoa040465 15084694

[17] Rapin N Bagger FO Jendholm J Mora-Jensen H Krogh A Kohlmann A Comparing cancer vs normal gene expression profiles identifies new disease entities and common transcriptional programs in AML patients. Blood (2014) 123(6):894–904. 10.1182/blood-2013-02-485771 24363398

[18] Jaiswal S Fontanillas P Flannick J Manning A Grauman PV Mar BG Age-related clonal hematopoiesis associated with adverse outcomes. N Engl J Med (2014) 371(26):2488–98. 10.1056/NEJMoa1408617 25426837 PMC4306669

[19] Genovese G Kahler AK Handsaker RE Lindberg J Rose SA Bakhoum SF Clonal hematopoiesis and blood-cancer risk inferred from blood DNA sequence. N Engl J Med (2014) 371(26):2477–87. 10.1056/NEJMoa1409405 25426838 PMC4290021

[20] Xie M Lu C Wang J McLellan MD Johnson KJ Wendl MC Age-related mutations associated with clonal hematopoietic expansion and malignancies. Nat Med (2014) 20(12):1472–8. 10.1038/nm.3733 25326804 PMC4313872

[21] Bonnet D Dick JE. Human acute myeloid leukemia is organized as a hierarchy that originates from a primitive hematopoietic cell. Nat Med (1997) 3(7):730–7. 10.1038/nm0797-730 9212098

[22] Shlush LI Zandi S Mitchell A Chen WC Brandwein JM Gupta V Identification of pre-leukaemic haematopoietic stem cells in acute leukaemia. Nature (2014) 506(7488):328–33. 10.1038/nature13038 24522528 PMC4991939

[23] Jan M Snyder TM Corces-Zimmerman MR Vyas P Weissman IL Quake SR Clonal evolution of preleukemic hematopoietic stem cells precedes human acute myeloid leukemia. Sci Transl Med (2012) 4(149):149ra18. 10.1126/scitranslmed.3004315 22932223 PMC4045621

[24] Ding L Ley TJ Larson DE Miller CA Koboldt DC Welch JS Clonal evolution in relapsed acute myeloid leukaemia revealed by whole-genome sequencing. Nature (2012) 481(7382):506–10. 10.1038/nature10738 22237025 PMC3267864

[25] Kiessling R Klein E Wigzell H “Natural” killer cells in the mouse. I. Cytotoxic cells with specificity for mouse Moloney leukemia cells. Specificity and distribution according to genotype. Eur J Immunol (1975) 5(2):112–7. 10.1002/eji.1830050208 1234049

[26] Kiessling R Klein E Pross H Wigzell H. “Natural” killer cells in the mouse. II. Cytotoxic cells with specificity for mouse Moloney leukemia cells. Characteristics of the killer cell. Eur J Immunol (1975) 5(2):117–21. 10.1002/eji.1830050209 1086218

[27] Herberman RB Nunn ME Lavrin DH. Natural cytotoxic reactivity of mouse lymphoid cells against syngeneic acid allogeneic tumors. I. Distribution of reactivity and specificity. Int J Cancer (1975) 16(2):216–29. 10.1002/ijc.2910160205 50294

[28] Herberman RB Nunn ME Holden HT Lavrin DH. Natural cytotoxic reactivity of mouse lymphoid cells against syngeneic and allogeneic tumors. II. Characterization of effector cells. Int J Cancer (1975) 16(2):230–9. 10.1002/ijc.2910160205 1080480

[29] Spits H Artis D Colonna M Diefenbach A Di Santo JP Eberl G Innate lymphoid cells – a proposal for uniform nomenclature. Nat Rev Immunol (2013) 13(2):145–9. 10.1038/nri3365 23348417

[30] Eberl G Colonna M Di Santo JP McKenzie AN. Innate lymphoid cells. Innate lymphoid cells: a new paradigm in immunology. Science (2015) 348(6237):aaa6566. 10.1126/science.aaa6566 25999512 PMC5658207

[31] Gasteiger G Rudensky AY. Interactions between innate and adaptive lymphocytes. Nat Rev Immunol (2014) 14(9):631–9. 10.1038/nri3726 25132095 PMC4504695

[32] Cooper MA Fehniger TA Caligiuri MA. The biology of human natural killer-cell subsets. Trends Immunol (2001) 22(11):633–40. 10.1016/S1471-4906(01)02060-9 11698225

[33] Fauriat C Long EO Ljunggren HG Bryceson YT. Regulation of human NK-cell cytokine and chemokine production by target cell recognition. Blood (2010) 115(11):2167–76. 10.1182/blood-2009-08-238469 19965656 PMC2844017

[34] Fehniger TA Cooper MA Nuovo GJ Cella M Facchetti F Colonna M CD56bright natural killer cells are present in human lymph nodes and are activated by T cell-derived IL-2: a potential new link between adaptive and innate immunity. Blood (2003) 101(8):3052–7. 10.1182/blood-2002-09-2876 12480696

[35] Long EO Kim HS Liu D Peterson ME Rajagopalan S. Controlling natural killer cell responses: integration of signals for activation and inhibition. Annu Rev Immunol (2013) 31:227–58. 10.1146/annurev-immunol-020711-075005 23516982 PMC3868343

[36] Ljunggren HG Karre K. In search of the ‘missing self’: MHC molecules and NK cell recognition. Immunol Today (1990) 11(7):237–44. 10.1016/0167-5699(90)90097-S 2201309

[37] Campoli M Ferrone S HLA antigen changes in malignant cells: epigenetic mechanisms and biologic significance. Oncogene (2008) 27(45):5869–85. 10.1038/onc.2008.273 18836468 PMC2729106

[38] Seliger B Ritz U Ferrone S. Molecular mechanisms of HLA class I antigen abnormalities following viral infection and transformation. Int J Cancer (2006) 118(1):129–38. 10.1002/ijc.21312 16003759

[39] Ardolino M Azimi CS Iannello A Trevino TN Horan L Zhang L Cytokine therapy reverses NK cell anergy in MHC-deficient tumors. J Clin Invest (2014) 124(11):4781–94. 10.1172/JCI74337 25329698 PMC4347250

[40] Pende D Rivera P Marcenaro S Chang CC Biassoni R Conte R Major histocompatibility complex class I-related chain A and UL16-binding protein expression on tumor cell lines of different histotypes: analysis of tumor susceptibility to NKG2D-dependent natural killer cell cytotoxicity. Cancer Res (2002) 62(21):6178–86. 12414645

[41] Guerra N Tan YX Joncker NT Choy A Gallardo F Xiong N NKG2D-deficient mice are defective in tumor surveillance in models of spontaneous malignancy. Immunity (2008) 28(4):571–80. 10.1016/j.immuni.2008.02.016 18394936 PMC3528789

[42] Gasser S Orsulic S Brown EJ Raulet DH. The DNA damage pathway regulates innate immune system ligands of the NKG2D receptor. Nature (2005) 436(7054):1186–90. 10.1038/nature03884 15995699 PMC1352168

[43] Raulet DH Gasser S Gowen BG Deng W Jung H. Regulation of ligands for the NKG2D activating receptor. Annu Rev Immunol (2013) 31:413–41. 10.1146/annurev-immunol-032712-095951 23298206 PMC4244079

[44] Marcucci G Mrozek K Radmacher MD Garzon R Bloomfield CD. The prognostic and functional role of microRNAs in acute myeloid leukemia. Blood (2011) 117(4):1121–9. 10.1182/blood-2010-09-191312 21045193 PMC3056468

[45] Reikvam H Hatfield KJ Ersvaer E Hovland R Skavland J Gjertsen BT Expression profile of heat shock proteins in acute myeloid leukaemia patients reveals a distinct signature strongly associated with FLT3 mutation status – consequences and potentials for pharmacological intervention. Br J Haematol (2012) 156(4):468–80. 10.1111/j.1365-2141.2011.08960.x 22150087

[46] Martelli AM Evangelisti C Chiarini F McCubrey JA. The phosphatidylinositol 3-kinase/Akt/mTOR signaling network as a therapeutic target in acute myelogenous leukemia patients. Oncotarget (2010) 1(2):89–103. 10.18632/oncotarget.114 20671809 PMC2911128

[47] Soriani A Zingoni A Cerboni C Iannitto ML Ricciardi MR Di Gialleonardo V ATM-ATR-dependent up-regulation of DNAM-1 and NKG2D ligands on multiple myeloma cells by therapeutic agents results in enhanced NK-cell susceptibility and is associated with a senescent phenotype. Blood (2009) 113(15):3503–11. 10.1182/blood-2008-08-173914 19098271

[48] Ardolino M Zingoni A Cerboni C Cecere F Soriani A Iannitto ML DNAM-1 ligand expression on Ag-stimulated T lymphocytes is mediated by ROS-dependent activation of DNA-damage response: relevance for NK-T cell interaction. Blood (2011) 117(18):4778–86. 10.1182/blood-2010-08-300954 21406724

[49] El-Sherbiny YM Meade JL Holmes TD McGonagle D Mackie SL Morgan AW The requirement for DNAM-1, NKG2D, and NKp46 in the natural killer cell-mediated killing of myeloma cells. Cancer Res (2007) 67(18):8444–9. 10.1158/0008-5472.CAN-06-4230 17875681

[50] Lakshmikanth T Burke S Ali TH Kimpfler S Ursini F Ruggeri L NCRs and DNAM-1 mediate NK cell recognition and lysis of human and mouse melanoma cell lines in vitro and in vivo. J Clin Invest (2009) 119(5):1251–63. 10.1172/JCI36022 19349689 PMC2673866

[51] Koch J Steinle A Watzl C Mandelboim O. Activating natural cytotoxicity receptors of natural killer cells in cancer and infection. Trends Immunol (2013) 34(4):182–91. 10.1016/j.it.2013.01.003 23414611

[52] Sanchez-Correa B Morgado S Gayoso I Bergua JM Casado JG Arcos MJ Human NK cells in acute myeloid leukaemia patients: analysis of NK cell-activating receptors and their ligands. Cancer Immunol Immunother (2011) 60(8):1195–205. 10.1007/s00262-011-1050-2 21644031 PMC11028638

[53] Nowbakht P Ionescu MC Rohner A Kalberer CP Rossy E Mori L Ligands for natural killer cell-activating receptors are expressed upon the maturation of normal myelomonocytic cells but at low levels in acute myeloid leukemias. Blood (2005) 105(9):3615–22. 10.1182/blood-2004-07-2585 15657183

[54] Brandt CS Baratin M Yi EC Kennedy J Gao Z Fox B The B7 family member B7-H6 is a tumor cell ligand for the activating natural killer cell receptor NKp30 in humans. J Exp Med (2009) 206(7):1495–503. 10.1084/jem.20090681 19528259 PMC2715080

[55] Baychelier F Sennepin A Ermonval M Dorgham K Debre P Vieillard V. Identification of a cellular ligand for the natural cytotoxicity receptor NKp44. Blood (2013) 122(17):2935–42. 10.1182/blood-2013-03-489054 23958951

[56] Khaznadar Z Henry G Setterblad N Agaugue S Raffoux E Boissel N Acute myeloid leukemia impairs natural killer cells through the formation of a deficient cytotoxic immunological synapse. Eur J Immunol (2014) 44(10):3068–80. 10.1002/eji.201444500 25041786

[57] Veillette A NK cell regulation by SLAM family receptors and SAP-related adapters. Immunol Rev (2006) 214:22–34. 10.1111/j.1600-065X.2006.00453.x 17100873

[58] Street SE Cretney E Smyth MJ. Perforin and interferon-gamma activities independently control tumor initiation, growth, and metastasis. Blood (2001) 97(1):192–7. 10.1182/blood.V97.1.192 11133760

[59] Street SE Trapani JA MacGregor D Smyth MJ. Suppression of lymphoma and epithelial malignancies effected by interferon gamma. J Exp Med (2002) 196(1):129–34. 10.1084/jem.20020063 12093877 PMC2194011

[60] Smyth MJ Swann J Cretney E Zerafa N Yokoyama WM Hayakawa Y. NKG2D function protects the host from tumor initiation. J Exp Med (2005) 202(5):583–8. 10.1084/jem.20050994 16129707 PMC2212868

[61] Smyth MJ Cretney E Kelly JM Westwood JA Street SE Yagita H Activation of NK cell cytotoxicity. Mol Immunol (2005) 42(4):501–10. 10.1016/j.molimm.2004.07.034 15607806

[62] Smyth MJ Takeda K Hayakawa Y Peschon JJ van den Brink MR Yagita H Nature’s TRAIL – on a path to cancer immunotherapy. Immunity (2003) 18(1):1–6. 10.1016/S1074-7613(02)00502-2 12530970

[63] Imai K Matsuyama S Miyake S Suga K Nakachi K. Natural cytotoxic activity of peripheral-blood lymphocytes and cancer incidence: an 11-year follow-up study of a general population. Lancet (2000) 356(9244):1795–9. 10.1016/S0140-6736(00)03231-1 11117911

[64] Martin-Fontecha A Thomsen LL Brett S Gerard C Lipp M Lanzavecchia A Induced recruitment of NK cells to lymph nodes provides IFN-gamma for T(H)1 priming. Nat Immunol (2004) 5(12):1260–5. 10.1038/ni1138 15531883

[65] O’Sullivan T Saddawi-Konefka R Vermi W Koebel CM Arthur C White JM Cancer immunoediting by the innate immune system in the absence of adaptive immunity. J Exp Med (2012) 209(10):1869–82. 10.1084/jem.20112738 22927549 PMC3457735

[66] Ruggeri L Capanni M Urbani E Perruccio K Shlomchik WD Tosti A Effectiveness of donor natural killer cell alloreactivity in mismatched hematopoietic transplants. Science (2002) 295(5562):2097–100. 10.1126/science.1068440 11896281

[67] Ruggeri L Mancusi A Capanni M Urbani E Carotti A Aloisi T Donor natural killer cell allorecognition of missing self in haploidentical hematopoietic transplantation for acute myeloid leukemia: challenging its predictive value. Blood (2007) 110(1):433–40. 10.1182/blood-2006-07-038687 17371948 PMC1896125

[68] Venstrom JM Pittari G Gooley TA Chewning JH Spellman S Haagenson M HLA-C-dependent prevention of leukemia relapse by donor activating KIR2DS1. N Engl J Med (2012) 367(9):805–16. 10.1056/NEJMoa1200503 22931314 PMC3767478

[69] Willemze R Rodrigues CA Labopin M Sanz G Michel G Socie G KIR-ligand incompatibility in the graft-versus-host direction improves outcomes after umbilical cord blood transplantation for acute leukemia. Leukemia (2009) 23(3):492–500. 10.1038/leu.2008.365 19151783 PMC7101531

[70] Brunstein CG Wagner JE Weisdorf DJ Cooley S Noreen H Barker JN Negative effect of KIR alloreactivity in recipients of umbilical cord blood transplant depends on transplantation conditioning intensity. Blood (2009) 113(22):5628–34. 10.1182/blood-2008-12-197467 19329778 PMC2689057

[71] Cichocki F Cooley S Davis Z DeFor TE Schlums H Zhang B CD56CD57NKG2C NK cell expansion is associated with reduced leukemia relapse after reduced intensity HCT. Leukemia (2015) 30(2):456–63. 10.1038/leu.2015.260 26416461 PMC4740203

[72] Miller JS Soignier Y Panoskaltsis-Mortari A McNearney SA Yun GH Fautsch SK Successful adoptive transfer and in vivo expansion of human haploidentical NK cells in patients with cancer. Blood (2005) 105(8):3051–7. 10.1182/blood-2004-07-2974 15632206

[73] Passweg JR Tichelli A Meyer-Monard S Heim D Stern M Kuhne T Purified donor NK-lymphocyte infusion to consolidate engraftment after haploidentical stem cell transplantation. Leukemia (2004) 18(11):1835–8. 10.1038/sj.leu.2403524 15457184

[74] Costello RT Sivori S Marcenaro E Lafage-Pochitaloff M Mozziconacci MJ Reviron D Defective expression and function of natural killer cell-triggering receptors in patients with acute myeloid leukemia. Blood (2002) 99(10):3661–7. 10.1182/blood.V99.10.3661 11986221

[75] Fauriat C Just-Landi S Mallet F Arnoulet C Sainty D Olive D Deficient expression of NCR in NK cells from acute myeloid leukemia: evolution during leukemia treatment and impact of leukemia cells in NCR^dull^ phenotype induction. Blood (2007) 109(1):323–30. 10.1182/blood-2005-08-027979 16940427

[76] Khaznadar Z Boissel N Agaugue S Henry G Cheok M Vignon M Defective NK cells in acute myeloid leukemia patients at diagnosis are associated with blast transcriptional signatures of immune evasion. J Immunol (2015) 195(6):2580–90. 10.4049/jimmunol.1500262 26246143

[77] Stringaris K Sekine T Khoder A Alsuliman A Razzaghi B Sargeant R Leukemia-induced phenotypic and functional defects in natural killer cells predict failure to achieve remission in acute myeloid leukemia. Haematologica (2014) 99(5):836–47. 10.3324/haematol.2013.087536 24488563 PMC4008119

[78] Fauriat C Moretta A Olive D Costello RT. Defective killing of dendritic cells by autologous natural killer cells from acute myeloid leukemia patients. Blood (2005) 106(6):2186–8. 10.1182/blood-2005-03-1270 15928036

[79] Kim R Emi M Tanabe K Arihiro K. Tumor-driven evolution of immunosuppressive networks during malignant progression. Cancer Res (2006) 66(11):5527–36. 10.1158/0008-5472.CAN-05-4128 16740684

[80] Catenacci DV Schiller GJ Myelodysplasic syndromes: a comprehensive review. Blood Rev (2005) 19(6):301–19. 10.1016/j.blre.2005.01.004 15885860

[81] Carlsten M Baumann BC Simonsson M Jadersten M Forsblom AM Hammarstedt C Reduced DNAM-1 expression on bone marrow NK cells associated with impaired killing of CD34+ blasts in myelodysplastic syndrome. Leukemia (2010) 24(9):1607–16. 10.1038/leu.2010.149 20613786

[82] Kiladjian JJ Bourgeois E Lobe I Braun T Visentin G Bourhis JH Cytolytic function and survival of natural killer cells are severely altered in myelodysplastic syndromes. Leukemia (2006) 20(3):463–70. 10.1038/sj.leu.2404080 16408099

[83] Epling-Burnette PK Bai F Painter JS Rollison DE Salih HR Krusch M Reduced natural killer (NK) function associated with high-risk myelodysplastic syndrome (MDS) and reduced expression of activating NK receptors. Blood (2007) 109(11):4816–24. 10.1182/blood-2006-07-035519 17341666 PMC1885518

[84] Kim Y Jekarl DW Kim J Kwon A Choi H Lee S Genetic and epigenetic alterations of bone marrow stromal cells in myelodysplastic syndrome and acute myeloid leukemia patients. Stem Cell Res (2015) 14(2):177–84. 10.1016/j.scr.2015.01.004 25665922

[85] Vasold J Wagner M Drolle H Deniffel C Kutt A Oostendorp R The bone marrow microenvironment is a critical player in the NK cell response against acute myeloid leukaemia in vitro. Leuk Res (2015) 39(2):257–62. 10.1016/j.leukres.2014.12.001 25542695

[86] Orange JS. Formation and function of the lytic NK-cell immunological synapse. Nat Rev Immunol (2008) 8(9):713–25. 10.1038/nri2381 19172692 PMC2772177

[87] Ritter AT Asano Y Stinchcombe JC Dieckmann NM Chen BC Gawden-Bone C Actin depletion initiates events leading to granule secretion at the immunological synapse. Immunity (2015) 42(5):864–76. 10.1016/j.immuni.2015.04.013 25992860 PMC4448150

[88] Li P Katirai F Zheng F Gong F. Recycling and reutilization of cytotoxic molecules, a new type of energy conservation of NK cells? Med Hypotheses (2011) 76(2):293–5. 10.1016/j.mehy.2010.10.027 21075541

[89] Le Dieu R Taussig DC Ramsay AG Mitter R Miraki-Moud F Fatah R Peripheral blood T cells in acute myeloid leukemia (AML) patients at diagnosis have abnormal phenotype and genotype and form defective immune synapses with AML blasts. Blood (2009) 114(18):3909–16. 10.1182/blood-2009-02-206946 19710498 PMC2773481

[90] Sanchez-Correa B Gayoso I Bergua JM Casado JG Morgado S Solana R Decreased expression of DNAM-1 on NK cells from acute myeloid leukemia patients. Immunol Cell Biol (2012) 90(1):109–15. 10.1038/icb.2011.15 21383766

[91] Marra J Greene J Hwang J Du J Damon L Martin T KIR and HLA genotypes predictive of low-affinity interactions are associated with lower relapse in autologous hematopoietic cell transplantation for acute myeloid leukemia. J Immunol (2015) 194(9):4222–30. 10.4049/jimmunol.1402124 25810393

[92] Chan CJ Martinet L Gilfillan S Souza-Fonseca-Guimaraes F Chow MT Town L The receptors CD96 and CD226 oppose each other in the regulation of natural killer cell functions. Nat Immunol (2014) 15(5):431–8. 10.1038/ni.2850 24658051

[93] Coles SJ Wang EC Man S Hills RK Burnett AK Tonks A CD200 expression suppresses natural killer cell function and directly inhibits patient anti-tumor response in acute myeloid leukemia. Leukemia (2011) 25(5):792–9. 10.1038/leu.2011.1 21274000 PMC3093357

[94] Salih HR Rammensee HG Steinle A. Cutting edge: down-regulation of MICA on human tumors by proteolytic shedding. J Immunol (2002) 169(8):4098–102. 10.4049/jimmunol.169.8.4098 12370336

[95] Waldhauer I Steinle A. Proteolytic release of soluble UL16-binding protein 2 from tumor cells. Cancer Res (2006) 66(5):2520–6. 10.1158/0008-5472.CAN-05-2520 16510567

[96] Schlecker E Fiegler N Arnold A Altevogt P Rose-John S Moldenhauer G Metalloprotease-mediated tumor cell shedding of B7-H6, the ligand of the natural killer cell-activating receptor NKp30. Cancer Res (2014) 74(13):3429–40. 10.1158/0008-5472.CAN-13-3017 24780758

[97] Boutet P Aguera-Gonzalez S Atkinson S Pennington CJ Edwards DR Murphy G Cutting edge: the metalloproteinase ADAM17/TNF-alpha-converting enzyme regulates proteolytic shedding of the MHC class I-related chain B protein. J Immunol (2009) 182(1):49–53. 10.4049/jimmunol.182.1.49 19109134

[98] Waldhauer I Goehlsdorf D Gieseke F Weinschenk T Wittenbrink M Ludwig A Tumor-associated MICA is shed by ADAM proteases. Cancer Res (2008) 68(15):6368–76. 10.1158/0008-5472.CAN-07-6768 18676862

[99] Liu G Atteridge CL Wang X Lundgren AD Wu JD. The membrane type matrix metalloproteinase MMP14 mediates constitutive shedding of MHC class I chain-related molecule A independent of A disintegrin and metalloproteinases. J Immunol (2010) 184(7):3346–50. 10.4049/jimmunol.0903789 20208009 PMC3191873

[100] Kaiser BK Yim D Chow IT Gonzalez S Dai Z Mann HH Disulphide-isomerase-enabled shedding of tumour-associated NKG2D ligands. Nature (2007) 447(7143):482–6. 10.1038/nature05768 17495932

[101] Huergo-Zapico L Gonzalez-Rodriguez AP Contesti J Gonzalez E Lopez-Soto A Fernandez-Guizan A Expression of ERp5 and GRP78 on the membrane of chronic lymphocytic leukemia cells: association with soluble MICA shedding. Cancer Immunol Immunother (2012) 61(8):1201–10. 10.1007/s00262-011-1195-z 22215138 PMC11029067

[102] Hilpert J Grosse-Hovest L Grunebach F Buechele C Nuebling T Raum T Comprehensive analysis of NKG2D ligand expression and release in leukemia: implications for NKG2D-mediated NK cell responses. J Immunol (2012) 189(3):1360–71. 10.4049/jimmunol.1200796 22730533

[103] Groh V Wu J Yee C Spies T. Tumour-derived soluble MIC ligands impair expression of NKG2D and T-cell activation. Nature (2002) 419(6908):734–8. 10.1038/nature01112 12384702

[104] Deng W Gowen BG Zhang L Wang L Lau S Iannello A Antitumor immunity. A shed NKG2D ligand that promotes natural killer cell activation and tumor rejection. Science (2015) 348(6230):136–9. 10.1126/science.1258867 25745066 PMC4856222

[105] Clayton A Mitchell JP Court J Linnane S Mason MD Tabi Z. Human tumor-derived exosomes down-modulate NKG2D expression. J Immunol (2008) 180(11):7249–58. 10.4049/jimmunol.180.11.7249 18490724

[106] Reiners KS Topolar D Henke A Simhadri VR Kessler J Sauer M Soluble ligands for NK cell receptors promote evasion of chronic lymphocytic leukemia cells from NK cell anti-tumor activity. Blood (2013) 121(18):3658–65. 10.1182/blood-2013-01-476606 23509156 PMC3643764

[107] Hedlund M Nagaeva O Kargl D Baranov V Mincheva-Nilsson L. Thermal- and oxidative stress causes enhanced release of NKG2D ligand-bearing immunosuppressive exosomes in leukemia/lymphoma T and B cells. PLoS One (2011) 6(2):e16899. 10.1371/journal.pone.0016899 21364924 PMC3045385

[108] Matta J Baratin M Chiche L Forel JM Cognet C Thomas G Induction of B7-H6, a ligand for the natural killer cell-activating receptor NKp30, in inflammatory conditions. Blood (2013) 122(3):394–404. 10.1182/blood-2013-01-481705 23687088

[109] Wang W Guo H Geng J Zheng X Wei H Sun R Tumor-released Galectin-3, a soluble inhibitory ligand of human NKp30, plays an important role in tumor escape from NK cell attack. J Biol Chem (2014) 289(48):33311–9. 10.1074/jbc.M114.603464 25315772 PMC4246088

[110] Cheng CL Hou HA Lee MC Liu CY Jhuang JY Lai YJ Higher bone marrow LGALS3 expression is an independent unfavorable prognostic factor for overall survival in patients with acute myeloid leukemia. Blood (2013) 121(16):3172–80. 10.1182/blood-2012-07-443762 23449638

[111] Sanchez-Correa B Bergua JM Campos C Gayoso I Arcos MJ Banas H Cytokine profiles in acute myeloid leukemia patients at diagnosis: survival is inversely correlated with IL-6 and directly correlated with IL-10 levels. Cytokine (2013) 61(3):885–91. 10.1016/j.cyto.2012.12.023 23357299

[112] Cifaldi L Prencipe G Caiello I Bracaglia C Locatelli F De Benedetti F Inhibition of natural killer cell cytotoxicity by interleukin-6: implications for the pathogenesis of macrophage activation syndrome. Arthritis Rheumatol (2015) 67(11):3037–46. 10.1002/art.39295 26251193

[113] Vredevoe DL Widawski M Fonarow GC Hamilton M Martinez-Maza O Gage JR. Interleukin-6 (IL-6) expression and natural killer (NK) cell dysfunction and anergy in heart failure. Am J Cardiol (2004) 93(8):1007–11. 10.1016/j.amjcard.2003.12.054 15081444

[114] Scheid C Young R McDermott R Fitzsimmons L Scarffe JH Stern PL. Immune function of patients receiving recombinant human interleukin-6 (IL-6) in a phase I clinical study: induction of C-reactive protein and IgE and inhibition of natural killer and lymphokine-activated killer cell activity. Cancer Immunol Immunother (1994) 38(2):119–26. 10.1007/s002620050044 8306367 PMC11038782

[115] Romero AI Thoren FB Brune M Hellstrand K. NKp46 and NKG2D receptor expression in NK cells with CD56dim and CD56bright phenotype: regulation by histamine and reactive oxygen species. Br J Haematol (2006) 132(1):91–8. 10.1111/j.1365-2141.2005.05842.x 16371024

[116] Sallmyr A Fan J Datta K Kim KT Grosu D Shapiro P Internal tandem duplication of FLT3 (FLT3/ITD) induces increased ROS production, DNA damage, and misrepair: implications for poor prognosis in AML. Blood (2008) 111(6):3173–82. 10.1182/blood-2007-05-092510 18192505

[117] Rassool FV Gaymes TJ Omidvar N Brady N Beurlet S Pla M Reactive oxygen species, DNA damage, and error-prone repair: a model for genomic instability with progression in myeloid leukemia? Cancer Res (2007) 67(18):8762–71. 10.1158/0008-5472.CAN-06-4807 17875717

[118] Mussai F De Santo C Abu-Dayyeh I Booth S Quek L McEwen-Smith RM Acute myeloid leukemia creates an arginase-dependent immunosuppressive microenvironment. Blood (2013) 122(5):749–58. 10.1182/blood-2013-01-480129 23733335 PMC3731930

[119] Curti A Aluigi M Pandolfi S Ferri E Isidori A Salvestrini V Acute myeloid leukemia cells constitutively express the immunoregulatory enzyme indoleamine 2,3-dioxygenase. Leukemia (2007) 21(2):353–5. 10.1038/sj.leu.2404485 17170728

[120] Della Chiesa M Carlomagno S Frumento G Balsamo M Cantoni C Conte R The tryptophan catabolite l-kynurenine inhibits the surface expression of NKp46- and NKG2D-activating receptors and regulates NK-cell function. Blood (2006) 108(13):4118–25. 10.1182/blood-2006-03-006700 16902152

[121] Curti A Pandolfi S Valzasina B Aluigi M Isidori A Ferri E Modulation of tryptophan catabolism by human leukemic cells results in the conversion of CD25- into CD25+ T regulatory cells. Blood (2007) 109(7):2871–7. 10.1182/blood-2006-07-036863 17164341

[122] Barao I Hanash AM Hallett W Welniak LA Sun K Redelman D Suppression of natural killer cell-mediated bone marrow cell rejection by CD4+CD25+ regulatory T cells. Proc Natl Acad Sci U S A (2006) 103(14):5460–5. 10.1073/pnas.0509249103 16567639 PMC1459377

[123] Gasteiger G Hemmers S Firth MA Le Floc’h A Huse M Sun JC IL-2-dependent tuning of NK cell sensitivity for target cells is controlled by regulatory T cells. J Exp Med (2013) 210(6):1167–78. 10.1084/jem.20122462 23650441 PMC3674692

[124] Lowdell MW Craston R Samuel D Wood ME O’Neill E Saha V Evidence that continued remission in patients treated for acute leukaemia is dependent upon autologous natural killer cells. Br J Haematol (2002) 117(4):821–7. 10.1046/j.1365-2141.2002.03495.x 12060116

[125] Funato T Harigae H Abe S Sasaki T. Assessment of drug resistance in acute myeloid leukemia. Expert Rev Mol Diagn (2004) 4(5):705–13. 10.1586/14737159.4.5.705 15347263

[126] Schiller GJ. Treatment of resistant disease. Leukemia (1998) 12(Suppl 1):S20–4. 9777890

[127] Shaffer BC Gillet JP Patel C Baer MR Bates SE Gottesman MM. Drug resistance: still a daunting challenge to the successful treatment of AML. Drug Resist Updat (2012) 15(1–2):62–9. 10.1016/j.drup.2012.02.001 22409994 PMC3348380

[128] Nanbakhsh A Visentin G Olive D Janji B Mussard E Dessen P miR-181a modulates acute myeloid leukemia susceptibility to natural killer cells. Oncoimmunology (2015) 4(12):e996475. 10.1080/2162402X.2014.996475 26587335 PMC4635878

[129] Diermayr S Himmelreich H Durovic B Mathys-Schneeberger A Siegler U Langenkamp U NKG2D ligand expression in AML increases in response to HDAC inhibitor valproic acid and contributes to allorecognition by NK-cell lines with single KIR-HLA class I specificities. Blood (2008) 111(3):1428–36. 10.1182/blood-2007-07-101311 17993609

[130] Poggi A Catellani S Garuti A Pierri I Gobbi M Zocchi MR. Effective in vivo induction of NKG2D ligands in acute myeloid leukaemias by all-*trans*-retinoic acid or sodium valproate. Leukemia (2009) 23(4):641–8. 10.1038/leu.2008.354 19151770

[131] Nanbakhsh A Pochon C Mallavialle A Amsellem S Bourhis JH Chouaib S. c-Myc regulates expression of NKG2D ligands ULBP1/2/3 in AML and modulates their susceptibility to NK-mediated lysis. Blood (2014) 123(23):3585–95. 10.1182/blood-2013-11-536219 24677544 PMC4198341

[132] Knight R. IMiDs: a novel class of immunomodulators. Semin Oncol (2005) 32(4 Suppl 5):S24–30. 10.1053/j.seminoncol.2005.06.018 16085014

[133] Maurer C Pflug N Bahlo J Kluth S Rhein C Cramer P Bendamustine and rituximab in combination with lenalidomide in patients with chronic lymphocytic leukemia. Eur J Haematol (2015). 10.1111/ejh.12714 26643449

[134] Ruan J Martin P Shah B Schuster SJ Smith SM Furman RR Lenalidomide plus rituximab as initial treatment for mantle-cell lymphoma. N Engl J Med (2015) 373(19):1835–44. 10.1056/NEJMoa1505237 26535512 PMC4710541

[135] Gribben JG Fowler N Morschhauser F. Mechanisms of action of lenalidomide in B-cell non-Hodgkin lymphoma. J Clin Oncol (2015) 33(25):2803–11. 10.1200/JCO.2014.59.5363 26195701 PMC5320950

[136] Zeidner JF Foster MC. Immunomodulatory drugs: IMiDs in acute myeloid leukemia (AML). Curr Drug Targets (2015). 10.2174/1389450116666150304104315 25738295

[137] Berg SL Cairo MS Russell H Ayello J Ingle AM Lau H Safety, pharmacokinetics, and immunomodulatory effects of lenalidomide in children and adolescents with relapsed/refractory solid tumors or myelodysplastic syndrome: a Children’s Oncology Group Phase I Consortium report. J Clin Oncol (2011) 29(3):316–23. 10.1200/JCO.2010.30.8387 21149673 PMC3056465

[138] Lioznov M El-Cheikh J Jr Hoffmann F Hildebrandt Y Ayuk F Wolschke C Lenalidomide as salvage therapy after allo-SCT for multiple myeloma is effective and leads to an increase of activated NK (NKp44(+)) and T (HLA-DR(+)) cells. Bone Marrow Transplant (2010) 45(2):349–53. 10.1038/bmt.2009.155 19584825

[139] Davies FE Raje N Hideshima T Lentzsch S Young G Tai YT Thalidomide and immunomodulatory derivatives augment natural killer cell cytotoxicity in multiple myeloma. Blood (2001) 98(1):210–6. 10.1182/blood.V98.1.210 11418482

[140] Bartlett JB Michael A Clarke IA Dredge K Nicholson S Kristeleit H Phase I study to determine the safety, tolerability and immunostimulatory activity of thalidomide analogue CC-5013 in patients with metastatic malignant melanoma and other advanced cancers. Br J Cancer (2004) 90(5):955–61. 10.1038/sj.bjc.6601579 14997189 PMC2410215

[141] Hayashi T Hideshima T Akiyama M Podar K Yasui H Raje N Molecular mechanisms whereby immunomodulatory drugs activate natural killer cells: clinical application. Br J Haematol (2005) 128(2):192–203. 10.1111/j.1365-2141.2004.05286.x 15638853

[142] Reddy N Hernandez-Ilizaliturri FJ Deeb G Roth M Vaughn M Knight J Immunomodulatory drugs stimulate natural killer-cell function, alter cytokine production by dendritic cells, and inhibit angiogenesis enhancing the anti-tumour activity of rituximab in vivo. Br J Haematol (2008) 140(1):36–45. 10.1111/j.1365-2141.2007.06841.x 17995965

[143] Mehta DS Wurster AL Grusby MJ. Biology of IL-21 and the IL-21 receptor. Immunol Rev (2004) 202:84–95. 10.1111/j.0105-2896.2004.00201.x 15546387

[144] Cantoni C Bottino C Vitale M Pessino A Augugliaro R Malaspina A NKp44, a triggering receptor involved in tumor cell lysis by activated human natural killer cells, is a novel member of the immunoglobulin superfamily. J Exp Med (1999) 189(5):787–96. 10.1084/jem.189.5.787 10049942 PMC2192947

[145] Farag SS Caligiuri MA Cytokine modulation of the innate immune system in the treatment of leukemia and lymphoma. Adv Pharmacol (2004) 51:295–318. 10.1016/S1054-3589(04)51013-X 15464915

[146] Nelson BH IL-2, regulatory T cells, and tolerance. J Immunol (2004) 172(7):3983–8. 10.4049/jimmunol.172.7.3983 15034008

[147] Szczepanski MJ Szajnik M Welsh A Foon KA Whiteside TL Boyiadzis M. Interleukin-15 enhances natural killer cell cytotoxicity in patients with acute myeloid leukemia by upregulating the activating NK cell receptors. Cancer Immunol Immunother (2010) 59(1):73–9. 10.1007/s00262-009-0724-5 19526239 PMC3721322

[148] Stroncek DF Melief CJ Castiello L Cesano A Cheever MA Civini S Highlights of the society for immunotherapy of cancer (SITC) 27th annual meeting. J Immunother Cancer (2013) 1(1):4 10.1186/2051-1426-1-4

[149] Boyiadzis M Memon S Carson J Allen K Szczepanski MJ Vance BA Up-regulation of NK cell activating receptors following allogeneic hematopoietic stem cell transplantation under a lymphodepleting reduced intensity regimen is associated with elevated IL-15 levels. Biol Blood Marrow Transplant (2008) 14(3):290–300. 10.1016/j.bbmt.2007.12.490 18275895

[150] Thiant S Yakoub-Agha I Magro L Trauet J Coiteux V Jouet JP Plasma levels of IL-7 and IL-15 in the first month after myeloablative BMT are predictive biomarkers of both acute GVHD and relapse. Bone Marrow Transplant (2010) 45(10):1546–52. 10.1038/bmt.2010.13 20190846

[151] Frederiksen KS Lundsgaard D Freeman JA Hughes SD Holm TL Skrumsager BK IL-21 induces in vivo immune activation of NK cells and CD8(+) T cells in patients with metastatic melanoma and renal cell carcinoma. Cancer Immunol Immunother (2008) 57(10):1439–49. 10.1007/s00262-008-0479-4 18286285 PMC2491425

[152] Burgess SJ Marusina AI Pathmanathan I Borrego F Coligan JE IL-21 down-regulates NKG2D/DAP10 expression on human NK and CD8+ T cells. J Immunol (2006) 176(3):1490–7. 10.4049/jimmunol.176.3.1490 16424177

[153] de Rham C Ferrari-Lacraz S Jendly S Schneiter G Dayer JM Villard J. The proinflammatory cytokines IL-2, IL-15 and IL-21 modulate the repertoire of mature human natural killer cell receptors. Arthritis Res Ther (2007) 9(6):R125. 10.1186/ar2336 18053164 PMC2246246

[154] Sivori S Cantoni C Parolini S Marcenaro E Conte R Moretta L IL-21 induces both rapid maturation of human CD34+ cell precursors towards NK cells and acquisition of surface killer Ig-like receptors. Eur J Immunol (2003) 33(12):3439–47. 10.1002/eji.200324533 14635054

[155] Davis ID Brady B Kefford RF Millward M Cebon J Skrumsager BK Clinical and biological efficacy of recombinant human interleukin-21 in patients with stage IV malignant melanoma without prior treatment: a phase IIa trial. Clin Cancer Res (2009) 15(6):2123–9. 10.1158/1078-0432.CCR-08-2663 19276257

[156] Denman CJ Senyukov VV Somanchi SS Phatarpekar PV Kopp LM Johnson JL Membrane-bound IL-21 promotes sustained ex vivo proliferation of human natural killer cells. PLoS One (2012) 7(1):e30264. 10.1371/journal.pone.0030264 22279576 PMC3261192

[157] Dahlberg CI Sarhan D Chrobok M Duru AD Alici E. Natural killer cell-based therapies targeting cancer: possible strategies to gain and sustain anti-tumor activity. Front Immunol (2015) 6:605. 10.3389/fimmu.2015.00605 26648934 PMC4663254

[158] Wiernik A Foley B Zhang B Verneris MR Warlick E Gleason MK Targeting natural killer cells to acute myeloid leukemia in vitro with a CD16 x 33 bispecific killer cell engager and ADAM17 inhibition. Clin Cancer Res (2013) 19(14):3844–55. 10.1158/1078-0432.CCR-13-0505 23690482 PMC3715574

[159] Gleason MK Verneris MR Todhunter DA Zhang B McCullar V Zhou SX Bispecific and trispecific killer cell engagers directly activate human NK cells through CD16 signaling and induce cytotoxicity and cytokine production. Mol Cancer Ther (2012) 11(12):2674–84. 10.1158/1535-7163.MCT-12-0692 23075808 PMC3519950

[160] Rothe A Sasse S Topp MS Eichenauer DA Hummel H Reiners KS A phase 1 study of the bispecific anti-CD30/CD16A antibody construct AFM13 in patients with relapsed or refractory Hodgkin lymphoma. Blood (2015) 125(26):4024–31. 10.1182/blood-2014-12-614636 25887777 PMC4528081

[161] Vallera DA Felices M McElmurry RT McCullar V Zhou X Schmohl J IL-15 trispecific killer engagers (TriKEs) make natural killer cells specific to CD33+ targets while also inducing in vivo expansion, and enhanced function. Clin Cancer Res (2016). 10.1158/1078-0432.CCR-15-2710 PMC4947440 26847056

[162] Grandjenette C Dicato M Diederich M. Bispecific antibodies: an innovative arsenal to hunt, grab and destroy cancer cells. Curr Pharm Biotechnol (2015) 16(8):670–83. 10.2174/1389201016666150505124037 25941884

[163] Childs RW Carlsten M. Therapeutic approaches to enhance natural killer cell cytotoxicity against cancer: the force awakens. Nat Rev Drug Discov (2015) 14(7):487–98. 10.1038/nrd4506 26000725

[164] Wischhusen J Friese MA Mittelbronn M Meyermann R Weller M HLA-E protects glioma cells from NKG2D-mediated immune responses in vitro: implications for immune escape in vivo. J Neuropathol Exp Neurol (2005) 64(6):523–8. 10.1093/jnen/64.6.523 15977644

[165] Malmberg KJ Levitsky V Norell H de Matos CT Carlsten M Schedvins K IFN-gamma protects short-term ovarian carcinoma cell lines from CTL lysis via a CD94/NKG2A-dependent mechanism. J Clin Invest (2002) 110(10):1515–23. 10.1172/JCI15564 12438449 PMC151808

[166] Nguyen S Beziat V Dhedin N Kuentz M Vernant JP Debre P HLA-E upregulation on IFN-gamma-activated AML blasts impairs CD94/NKG2A-dependent NK cytolysis after haplo-mismatched hematopoietic SCT. Bone Marrow Transplant (2009) 43(9):693–9. 10.1038/bmt.2008.380 19011664

[167] Kohrt HE Thielens A Marabelle A Sagiv-Barfi I Sola C Chanuc F Anti-KIR antibody enhancement of anti-lymphoma activity of natural killer cells as monotherapy and in combination with anti-CD20 antibodies. Blood (2014) 123(5):678–86. 10.1182/blood-2013-08-519199 24326534 PMC3907754

[168] Romagne F Andre P Spee P Zahn S Anfossi N Gauthier L Preclinical characterization of 1-7F9, a novel human anti-KIR receptor therapeutic antibody that augments natural killer-mediated killing of tumor cells. Blood (2009) 114(13):2667–77. 10.1182/blood-2009-02-206532 19553639 PMC2756126

[169] Benson DM Jr Bakan CE Zhang S Collins SM Liang J Srivastava S IPH2101, a novel anti-inhibitory KIR antibody, and lenalidomide combine to enhance the natural killer cell versus multiple myeloma effect. Blood (2011) 118(24):6387–91. 10.1182/blood-2011-06-360255 22031859 PMC3490103

[170] Binyamin L Alpaugh RK Hughes TL Lutz CT Campbell KS Weiner LM. Blocking NK cell inhibitory self-recognition promotes antibody-dependent cellular cytotoxicity in a model of anti-lymphoma therapy. J Immunol (2008) 180(9):6392–401. 10.4049/jimmunol.180.9.6392 18424763 PMC2810560

[171] Vahlne G Lindholm K Meier A Wickstrom S Lakshmikanth T Brennan F In vivo tumor cell rejection induced by NK cell inhibitory receptor blockade: maintained tolerance to normal cells even in the presence of IL-2. Eur J Immunol (2010) 40(3):813–23. 10.1002/eji.200939755 20039300

[172] Sola C Andre P Lemmers C Fuseri N Bonnafous C Blery M Genetic and antibody-mediated reprogramming of natural killer cell missing-self recognition in vivo. Proc Natl Acad Sci U S A (2009) 106(31):12879–84. 10.1073/pnas.0901653106 19561305 PMC2722344

[173] Korde N Carlsten M Lee MJ Minter A Tan E Kwok M A phase II trial of pan-KIR2D blockade with IPH2101 in smoldering multiple myeloma. Haematologica (2014) 99(6):e81–3. 10.3324/haematol.2013.103085 24658821 PMC4040899

[174] Vey N Bourhis JH Boissel N Bordessoule D Prebet T Charbonnier A A phase 1 trial of the anti-inhibitory KIR mAb IPH2101 for AML in complete remission. Blood (2012) 120(22):4317–23. 10.1182/blood-2012-06-437558 23002117

[175] Benson DM Jr Hofmeister CC Padmanabhan S Suvannasankha A Jagannath S Abonour R A phase 1 trial of the anti-KIR antibody IPH2101 in patients with relapsed/refractory multiple myeloma. Blood (2012) 120(22):4324–33. 10.1182/blood-2012-06-438028 23033266 PMC3507143

[176] Benson DM Jr Cohen AD Jagannath S Munshi NC Spitzer G Hofmeister CC A phase I trial of the anti-KIR antibody IPH2101 and lenalidomide in patients with relapsed/refractory multiple myeloma. Clin Cancer Res (2015) 21(18):4055–61. 10.1158/1078-0432.CCR-15-0304 25999435 PMC4573800

[177] van der Veer MS de Weers M van Kessel B Bakker JM Wittebol S Parren PW Towards effective immunotherapy of myeloma: enhanced elimination of myeloma cells by combination of lenalidomide with the human CD38 monoclonal antibody daratumumab. Haematologica (2011) 96(2):284–90. 10.3324/haematol.2010.030759 21109694 PMC3031697

[178] Huang BY Zhan YP Zong WJ Yu CJ Li JF Qu YM The PD-1/B7-H1 pathway modulates the natural killer cells versus mouse glioma stem cells. PLoS One (2015) 10(8):e0134715. 10.1371/journal.pone.0134715 26266810 PMC4534134

[179] Wiesmayr S Webber SA Macedo C Popescu I Smith L Luce J Decreased NKp46 and NKG2D and elevated PD-1 are associated with altered NK-cell function in pediatric transplant patients with PTLD. Eur J Immunol (2012) 42(2):541–50. 10.1002/eji.201141832 22105417 PMC3607363

[180] Benson DM Jr Bakan CE Mishra A Hofmeister CC Efebera Y Becknell B The PD-1/PD-L1 axis modulates the natural killer cell versus multiple myeloma effect: a therapeutic target for CT-011, a novel monoclonal anti-PD-1 antibody. Blood (2010) 116(13):2286–94. 10.1182/blood-2010-02-271874 20460501 PMC3490105

[181] Berthon C Driss V Liu J Kuranda K Leleu X Jouy N In acute myeloid leukemia, B7-H1 (PD-L1) protection of blasts from cytotoxic T cells is induced by TLR ligands and interferon-gamma and can be reversed using MEK inhibitors. Cancer Immunol Immunother (2010) 59(12):1839–49. 10.1007/s00262-010-0909-y 20814675 PMC2945474

[182] Bellucci R Martin A Bommarito D Wang K Hansen SH Freeman GJ Interferon-gamma-induced activation of JAK1 and JAK2 suppresses tumor cell susceptibility to NK cells through upregulation of PD-L1 expression. Oncoimmunology (2015) 4(6):e1008824 10.1080/2162402X.2015.1008824 26155422 PMC4485824

[183] Töpfer K Cartellieri M Michen S Wiedemuth R Muller N Lindemann D DAP12-based activating chimeric antigen receptor for NK cell tumor immunotherapy. J Immunol (2015) 194(7):3201–12. 10.4049/jimmunol.1400330 25740942

[184] Suerth JD Morgan MA Kloess S Heckl D Neudorfl C Falk CS Efficient generation of gene-modified human natural killer cells via alpharetroviral vectors. J Mol Med (Berl) (2015) 94(1):83–93. 10.1007/s00109-015-1327-6 26300042

[185] Oyer JL Igarashi RY Kulikowski AR Colosimo DA Solh MM Zakari A Generation of highly cytotoxic natural killer cells for treatment of acute myelogenous leukemia using a feeder-free, particle-based approach. Biol Blood Marrow Transplant (2015) 21(4):632–9. 10.1016/j.bbmt.2014.12.037 25576425

[186] Boissel L Betancur-Boissel M Lu W Krause DS Van Etten RA Wels WS Retargeting NK-92 cells by means of CD19- and CD20-specific chimeric antigen receptors compares favorably with antibody-dependent cellular cytotoxicity. Oncoimmunology (2013) 2(10):e26527. 10.4161/onci.26527 24404423 PMC3881109

[187] Baek HJ Kim JS Yoon M Lee JJ Shin MG Ryang DW Ex vivo expansion of natural killer cells using cryopreserved irradiated feeder cells. Anticancer Res (2013) 33(5):2011–9. 23645750

